# Metabolomic Analysis of Biosynthesis Mechanism of ε-Polylysine Produced by *Streptomyces diastatochromogenes*

**DOI:** 10.3389/fbioe.2021.698022

**Published:** 2021-07-30

**Authors:** Ziyuan Wang, Fengzhu Guo, Tianyu Dong, Zhilei Tan, Mohamed Abdelraof, Zichen Wang, Jiandong Cui, Shiru Jia

**Affiliations:** ^1^State Key Laboratory of Food Nutrition and Safety, Laboratory of Industrial Fermentation Microbiology, Ministry of Education, College of Biotechnology, Tianjin University of Science and Technology, Tianjin, China; ^2^Tianjin Beiyang Baichuan Biotechnology Co., Ltd., Tianjin, China; ^3^Genetic Engineering and Biotechnology Research Division, National Research Centre, Dokki, Giza, Egypt

**Keywords:** ε-polylysine, *Streptomyces diastatochromogenes*, biosynthesis mechanism, metabolomics, high-yield strain

## Abstract

ε-Polylysine (ε-PL), a natural preservative with broad-spectrum antimicrobial activity, has been widely used as a green food additive, and it is now mainly produced by *Streptomyces* in industry. In the previous study, strain 6#-7 of high-yield ε-PL was obtained from the original strain TUST by mutagenesis. However, the biosynthesis mechanism of ε-PL in 6#-7 is still unclear. In this study, the metabolomic analyses of the biosynthesis mechanism of ε-PL in both strains are investigated. Results show that the difference in metabolisms between TUST and 6#-7 is significant. Based on the results of both metabolomic and enzymatic activities, a metabolic regulation mechanism of the high-yield strain is revealed. The transport and absorption capacity for glucose of 6#-7 is improved. The enzymatic activity benefits ε-PL synthesis, such as pyruvate kinase and aspartokinase, is strengthened. On the contrary, the activity of homoserine dehydrogenase in the branched-chain pathways is decreased. Meanwhile, the increase of trehalose, glutamic acid, etc. makes 6#-7 more resistant to ε-PL. Thus, the ability of the mutagenized strain 6#-7 to synthesize ε-PL is enhanced, and it can produce more ε-PLs compared with the original strain. For the first time, the metabolomic analysis of the biosynthesis mechanism of ε-PL in the high-yield strain 6#-7 is investigated, and a possible mechanism is then revealed. These findings provide a theoretical basis for further improving the production of ε-PL.

## Introduction

ε-Polylysine (ε-PL) is one of the two amino acid homopolymers that have been found in nature. It consists of 25–35 l-lysine residues linked through the α-carboxyl and ε-amino groups (Supplementary Figure 1) (El-Sersy et al., 2012), and it is produced by microbial metabolism (mainly by actinomycetes) (Shukla et al., 2012). It has various functions such as antibacterial (Shima et al., 1984) and antiphage activities (Shima et al., 1982). In addition, due to its good biocompatibility, it can be used as a matrix cross-linker agent for cardiovascular surgery (Fusaro et al., 2020). Also, the mouse-feeding experiments verified that the polymer has almost no acute or chronic toxicity (Hiraki et al., 2003). ε-PL was first found in the fermentation broth of *Streptomyces* sp. NBRC 14147 (Shima and Sakai, 1977). Currently, ε-PL, which is produced by mutant strains of NBRC 14147, had been commercially approved as a food additive (Oppermann-Sanio and Steinbuchel, 2002).

To improve the yield of ε-PL, Zong et al. (2012) obtained a mutagenized strain of *Streptomyces albulus* and the yield of ε-PL increased from 0.40 to 1.59 g/L. Wang et al. (2016) found that the enhancement of gentamicin resistance was related to the promotion of ε-PL production in the recombinants, and thus by three successive rounds of genome shuffling through protoplast fusion combined with increasing concentration of gentamicin for selection, the yield of ε-PL in *S. albulus* AG3-28 was improved to 3.43 g/L, which was 49.1% greater than the starting strain. Shima et al. reported that l-lysine can be directly utilized to produce ε-PL in biosynthesis (Shima et al., 1983). By investigating the feedback of aspartate kinase, Hamano et al. obtained a high-yield ε-PL-producing strain (M68V) (Hamano et al., 2007). By knocking out the homoserine dehydrogenase (HSD) gene in *Streptomyces clavuligerus*, (Yilmaz and Çaydasi, 2008) obtained a mutagenized strain with a 4.3-fold ε-PL yield compared with the original strain. Vanooyen et al. (2012) found that reducing the activity of citrate synthase can buffer the increase in the substrate and thus obtained a high-yield lysine-producing strain of *Corynebacterium glutamicum*.

The mechanism of the high production of ε-PL was also studied. The mixture of glucose and glycerol as a carbon source can promote the glycolytic pathway in *S. albulus* M-Z18 that benefits the energy consumption and provides sufficient precursors for ε-PL production (Zeng et al., 2017). Zhang et al. (2018) found the synthesis of oxaloacetate (Oaa) from phosphoenolpyruvate increased that provided more l-lysine and ultimately led to higher ε-PL productivity. The enhanced transcription of genes related to the central carbon metabolism, l-lysine synthesis, and cell respiration contributes to the increased production of ε-PL when using mixed carbon sources (Zeng et al., 2019). Liu et al. (2019) conducted a comparative proteomic analysis between the high-yield ε-PL mutant strain *S. albulus* SS-62 and the original strain M-Z18, and the results indicated that in strain SS-62 the synthesis of ε-PL precursor increased and the related transcriptional regulation and translation were tuned, which provided a better intracellular metabolic environment for the synthesis of ε-PL in strain SS-62. Recently, the metabolomic analysis of ε-PL bioproduction in a mutant *S. albulus* strain SAR 14-116 obtained through ARTP mutagenesis has been reported (Xiang et al., 2020).

In our previous study, a high-yield ε-PL mutant *Streptomyces diastatochromogenes* strain (6#-7) was obtained from the original strain TUST which was isolated from the soil samples of Hainan Island. Specifically, strain 6#-7 was obtained by incubating with 0.8 g/L nitrosoguanidine (NTG) for 45 min, followed by the UV irradiation for 45 s under magnetic stirring, and then through a series of screening, such as resistant plate, methylene blue plate, and shake flask screening (Song et al., 2014). At present, the biosynthesis mechanism of ε-PL by the high-yield strain 6#-7 is still unclear. As mentioned earlier, although many studies focus on the mechanism of high yield of ε-PL in *S. albulus* sp., the metabonomic analysis of the biosynthetic mechanism of ε-PL produced by *S. diastatochromogenes* has seldom been reported. Although *S. albulus* and *S. diastatochromogenes* are of different species from the same genera, their characteristics and metabolism may vary a lot (Georgiev et al., 2011). Thus, it is necessary and meaningful to reveal the biosynthesis mechanism of ε-PL in the high-yield strain 6#-7. In this study, the starting strain TUST and the high-yield mutant strain 6#-7 are investigated to reveal the biosynthesis mechanism of ε-PL. Through the multilevel analysis, the biosynthesis mechanism of the high-yield mutants is elucidated at the aspects of metabolic regulation and enzymology.

## Materials and Methods

### Strains, Medium, and Culture Conditions

The strain was inoculated into Bennett's slant medium and cultured at a temperature of 30°C with a humidity of 50% for 5–7 days to harvest gray spores. A ring of spores was picked from Bennett's slope and inserted into 100 ml of M3G seed medium. The flasks were incubated at 30°C and 180 rpm on a shaker for 30 h. Subsequently, the seed culture was transferred to another 500-ml flask containing 100 ml of fermentation medium at an inoculation amount of 6.4% (v/v) and was cultured at 30°C and 180 rpm for 72 h.

Bennett's solid medium (pH 7.7) is comprised of 10 g/L glucose, 1 g/L beef extract, 2 g/L peptone, 1 g/L yeast extract, and 20 g/L agar. The M3G seed and fermentation medium contained 10 g/L ammonium sulfate, 1.36 g/L potassium dihydrogen phosphate, 0.8 g/L dipotassium phosphate, and 5 g/L yeast extract, and the initial pH was adjusted to 7.2 with ammonium hydroxide solution.

To evaluate the effects on the synthesis of ε-PL by high-yield strains, the fermentation parameters, such as pH, biomass, residual sugar (RG), and ε-PL yield, were measured every 24 h. The pH value was measured by using an FE20 pH meter (METTLER TOLEDO, Shanghai). The biomass concentrations were calculated from the dry cell weight. In brief, 8-ml culture aliquots were taken every 24 h and centrifuged at 6,000×*g* for 10 min, and the obtained residues were washed three times with sterile water and finally dried at 95°C to constant weight. The corresponding biomass was calculated from the dried samples. The supernatant was diluted 50 times with distilled water, and the residual glucose (i.e., extracellular) concentration in the fermentation broth was measured by an SBA-40E biosensor analyzer (Biology Institute of Shandong Academy of Sciences, Shandong, China). The ε-PL yield was determined by the spectrophotometer (Thermo Fisher, China). The fermentation broth cultured for a certain period was centrifuged at 8,000×*g* for 5 min, and the supernatant was appropriately diluted. Later, the diluted solution was mixed with 1 mM methyl orange solution at a ratio of 1:1, shaken at 30°C and 140 rpm for 30 min, and centrifuged at 4,000×*g* for 15 min. Of note, 1 ml of the supernatant was diluted to 50 ml with 0.1 M Na_2_HPO_4_-NaH_2_PO_4_ buffer (pH 6.6), and then the absorbance at 465 nm was measured (i.e., a pure buffer was used as a blank control).

### Gas Chromatography–Mass Spectrometry (GC-MS)-Based Metabolomic Testing

Prior to the GC–MS analysis, the sample was derivatized by the method proposed by Bo et al. (2014). In brief, 50 μL of methoxy ammonium hydrochloride/pyridine solution (20 mg/ml) was added to the freeze-dried sample, vortexed to fully dissolve it, placed in a 40°C water bath, and shaken for 80 min. Then, 80 μL of N-methyl-N-(trimethylsilyl) trifluoroacetamide (MSTFA) was added, vortexed, and placed back in the water bath for another 80 min. Finally, after centrifuged at 10,000×*g* for 5 min, 100-μL supernatants of the derivatized sample were taken and placed at room temperature for 2 h.

The GC–MS system consisted of an Agilent 7890A GC system and an Agilent 5795C quadrupole mass selective detector (Agilent Technologies, Palo Alto, CA, USA). GC was performed on an HP-5 column (60 m × 0.250 mm × 0.25 μm, Agilent Technologies). A total of 1 μL of the derivatized sample was injected with a split ratio of 1:10. The temperatures of injector, GC interface, and ion source were set at 280, 250, and 280°C, respectively. The oven temperature was initially controlled at 70°C for 2 min, increased to 290°C with a gradient of 5°C/min, kept at 290°C for another 3 min, and finally dropped to 70°C. The carrier gas was helium at a constant flow of 1 ml/min. Mass spectra were recorded at two spectra per second with an m/z 50–800 scanning range.

Both identification and quantification of the mass spectral peaks were performed by MESCHEM software (Agilent Technologies). Peak deconvolution was previously performed by AMDIS-32. The NIST MS standard reference databases 2.0 were used for the identification of metabolites. The relative content of each metabolite was calculated by dividing its GC peak area by that of the internal standard (Liu et al., 2015). Also, the data were normalized as a percentage of that of the original strain for further analyses.

### Data Analysis

The mathematical model and the reliability verification of the model are important guarantees for the accuracy of later data analysis. Also, cross-validation is a practical and accurate method to test whether the model is effective (Han and Yuan, 2009). Therefore, the principal component analysis (PCA) and the partial least squares analysis (PLSA) by SIMCA-P 11.5 were used. The spectra were reconstructed by peak area integration (Lv et al., 2016), and after being centralized and orthogonal signal correction (OSC), the PCA was performed. The raw data were converted from multiple metrics into comprehensive metrics, and the clustering effect was previewed by a score plot. Besides, the loading plot was used to find biomarkers. Then, the PLSA was used for the verification analysis. Also, the sample grouping, biomarker, and contribution rate of the metabolites were verified by the score plot, the loading plot, and the variable importance plot (VIP), respectively. The data were intercepted for the hierarchical cluster analysis (HCA) for metabolites with a threshold greater than 0.5. Meanwhile, the data were statistically analyzed by SPPSS (SPSS Inc., Chicago, USA) software version 20.0.

### Determination of Enzyme Activity

Aspartokinase (ASK) activity was detected by measuring the amount of aspartyl-β-hydroxamate formed as described by Shiio and Miyajima (Shiio and Miyajima, 1969). The assay mixture contained in a 0.5-ml volume is as follows: 100 mM Tris–H_2_SO_4_ (pH 7.0), 600 mM (NH_4_)_2_SO_4_, 600 mM hydroxylamine–KOH (pH 7.0), 10 mM ATP, 10 mM MgSO_4_, 10 mM aspartic acid–KOH (pH 7.0), and 40 μg/ml ASK. The reaction mixtures were incubated for 10 min at 30°C, and 0.75 ml of ferric chloride solution (i.e., 10% FeCl_3_·6H_2_O and 3.4% trichloroacetic acid in 0.7 mol/L HCl) was added. After centrifugation, the A_540_ was measured in the supernatant. The background activity was measured in the absence of aspartic acid. Kinetic assays were performed under conditions identical to those described earlier, except that the enzyme concentration (i.e., 20 μg/ml) was reduced to enable the measurement of steady-state kinetic parameters. All assays were carried out under linear conditions. The amount of aspartyl-β-hydroxamate was calculated from the molar extinction coefficient of 600. One unit of enzyme activity is defined as the amount of enzyme catalyzing the formation of 1 μmol aspartyl β-hydroxamate per minute at 30°C. The protein concentrations were determined using a Bio-Rad Protein Assay Kit (Bio-Rad, Hercules, CA, USA). Bovine serum albumin was used as the standard protein.

The activity of HSD in both strains was measured in the reverse reaction by determining the initial rate of increase of A_340_ at room temperature (Hamano et al., 2007). The standard assay mixture contained 0.2 ml of 500 mM Tris–HCl buffer containing 5 mM ethylenediaminetetraacetic acid (EDTA), pH 8.4; 0.1 ml of 100 mM l-homoserine, pH 7.0; 0.1 ml of 4 mM triphosphopyridine nucleotide disodium salt (NADP-Na_2_) solution, 0.5 ml of distilled water, and 0.1 ml of cell-free extract or purified enzyme. For crude extracts, the absorbance at 340 nm was measured against a blank control containing all the components except the substrate. The specific activity of HSD was expressed as micromoles of nicotinamide adenine dinucleotide phosphate (NADPH) formed per minute per milligram of protein.

Polylysine synthetase (PLS) activity follows the method proposed by Kawai et al. (2003). The activity of enzyme preparation to synthesize ε-PL from l-lysine is defined as ε-PL-synthesizing activity. The reaction mixture for the assay of ε-PL-synthesizing activity contained 74 kBq/ml l-lysine, 5 mM ATP, 5 mM MgCl_2_, 100 mM Tris–HCl buffer (pH 8.0), 5 mM dithiothreitol (DTT), and 3 μL of enzyme preparation (i.e., 3–15 μg of protein) in a final volume of 15 μL. The reaction was carried out at 30°C for 30 min. Of note, 10 μL of the reaction mixture was taken and subjected to the 0.25% sodium tungstate dihydrate (W) and 5% trichloroacetic acid (TCA) treatment, followed by the radioactivity assay of ε-PL in a similar way as described earlier.

The activities of glucose-6-phosphate dehydrogenase (G6PDH), phosphoenolpyruvate carboxylase (PEPC), succinate dehydrogenase (SDH), pyruvate kinase (PK), and hexokinase (HK) were determined using the kit from Suzhou Comin Biotechnology Co., Ltd., China.

## Results and Discussion

### Comparison of Fermentation Performance Between the Original Strain TUST and the High-Yield Strain 6#-7

The difference between the original strain TUST and the high-yield strain 6#-7 in the fermentation performance of ε-PL production was compared by shake flask fermentation, which provided a foundation of the data for the exploration of ε-PL biosynthesis mechanisms.

**Figure 1:**
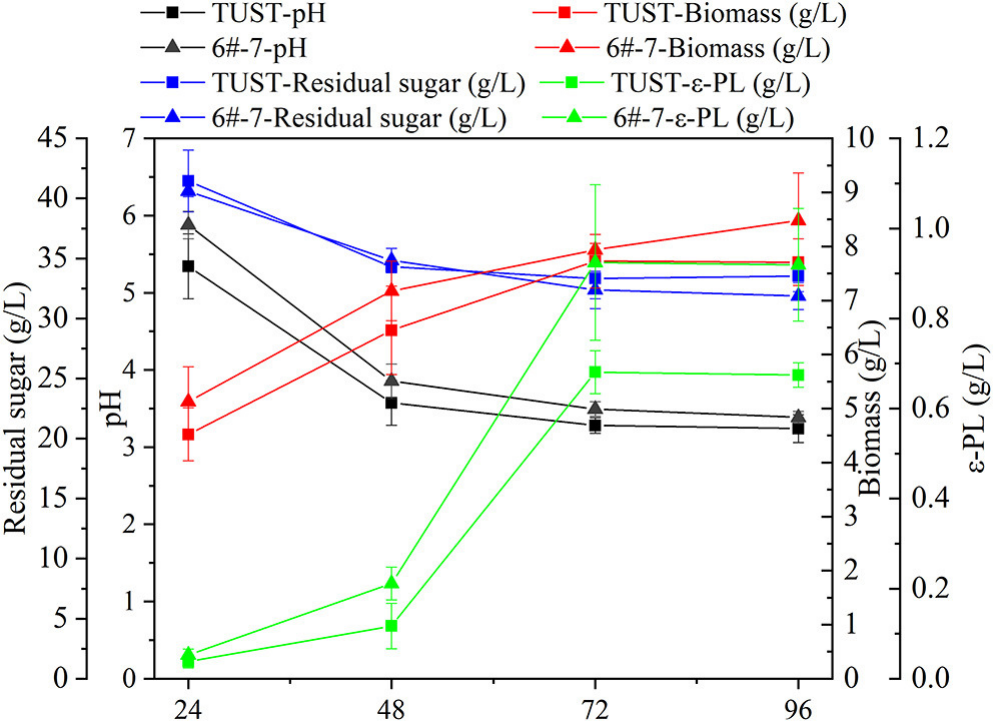
Fermentation performance of the strains.

The fermentation characteristics of the wild-type strain TUST and the mutant strain 6#-7 are shown in Figure 1. With the increase in fermentation time, the pH value of the fermentation broth showed a downward trend due to the production of primary metabolic organic acid. During 24–48 h, the pH value decreased obviously in the early stages of the fermentation due to the accumulation of various acids (Table 1; Supplementary Table 1), while the downward trend became slower during 48–72 h. In addition, the pH value of the fermentation broth of strain 6#-7 was slightly larger than that of the starting strain TUST during the whole fermentation process. As for RG, it also showed a downward trend indicating that both strains were continuously consuming the carbon source. Also, there was no significant difference in the sugar consumption rate between the two strains. In addition, the biomass of strain TUST reached a maximum of 7.72 g/L at 72 h and then decreased. However, the biomass of strain 6#-7 kept increasing and reached 8.48 g/L at 96 h. During 24–48 h, there was less difference in the amount of ε-PL between the two strains, while in the middle and late stages of fermentation 6#-7 produced more ε-PL than that of TUST. For both strains, the amount of ε-PL reached a maximum at 72 h, and the yield of strain 6#-7 reached 0.92 g/L, which was much larger than the original strain.

**Table 1:** Relative contents of intracellular metabolite in both strains identified by the gas chromatography–mass spectrometry (GC–MS) analysis.

**Metabolites**	**TUST**	**6#-7**						
	**12 h**	**24 h**	**36 h**	**48 h**	**12 h**	**24 h**	**36 h**	**48 h**
l-Aspartic acid^a^	ND	ND	ND	ND	0.11 ± 0.02	ND	0.29 ± 0.04	0.01 ± 0.01
l-Aspartic acid^b^	ND	ND	ND	ND	0.40 ± 0.09	0.21 ± 0.01	1.15 ± 0.09	0.03 ± 0.01
l-Asparagine	ND	0.63 ± 0.20	0.21 ± 0.03	0.22 ± 0.03	0.05 ± 0.01	1.15 ± 0.09	1.35 ± 0.05	0.26 ± 0.01
l-Lysine	0.06 ± 0.01	0.03 ± 0.01	0.16 ± 0.05	0.11 ± 0.05	0.04 ± 0.01	0.05 ± 0.01	0.33 ± 0.02	0.21 ± 0.03
d-Glucose	55.07 ± 1.74	109.51 ± 17.11	13.12 ± 1.97	4.70 ± 0.80	145.32 ± 4.04	80.47 ± 3.69	63.76 ± 2.42	0.38 ± 0.03
Maltose^a^	0.06 ± 0.03	0.06 ± 0.01	0.07 ± 0.02	0.14 ± 0.01	ND	ND	ND	ND
Maltose^b^	0.10 ± 0.04	0.16 ± 0.03	0.04 ± 0.02	0.05 ± 0.01	0.09 ± 0.03	0.08 ± 0.01	0.09 ± 0.01	0.06 ± 0.01
d-(+)-Trehalose	1.07 ± 0.15	11.12 ± 0.91	3.58 ± 0.55	2.07 ± 0.26	0.99 ± 0.10	35.58 ± 1.92	70.92 ± 2.73	1.40 ± 0.13
Hexadecenoic acid^a^	ND	ND	0.13 ± 0.01	0.25 ± 0.04	ND	ND	ND	0.07 ± 0.01
Hexadecenoic acid^b^	15.67 ± 0.59	7.32 ± 0.52	2.91 ± 0.18	2.76 ± 0.23	4.35 ± 0.45	2.88 ± 0.39	2.83 ± 0.03	2.70 ± 0.28
Octadecanoic acid^a^	16.18 ± 0.42	8.34 ± 2.40	8.85 ± 0.70	7.80 ± 0.11	12.11 ± 0.50	8.86 ± 0.50	8.26 ± 0.40	7.88 ± 0.27
Octadecanoic acid^b^	9.89 ± 0.76	5.16 ± 1.76	4.43 ± 1.20	3.69 ± 0.40	6.69 ± 0.29	3.98 ± 0.60	3.89 ± 0.14	3.74 ± 0.39
l-Norvaline	0.01 ± 0.01	0.01 ± 0.01	0.01 ± 0.01	0.01 ± 0.01	ND	0.01 ± 0.01	0.01 ± 0.01	0.01 ± 0.01
Sedoheptulose	ND	ND	0.01 ± 0.01	ND	ND	ND	0.01 ± 0.01	0.01 ± 0.01
1-Dodecanol	0.06 ± 0.01	0.04 ± 0.01	0.05 ± 0.01	0.04 ± 0.01	0.06 ± 0.01	0.06 ± 0.02	0.05 ± 0.01	0.03 ± 0.01
Acetamide^a^	0.07 ± 0.01	0.03 ± 0.01	0.05 ± 0.01	0.04 ± 0.01	0.05 ± 0.01	0.05 ± 0.01	0.09 ± 0.01	0.04 ± 0.01
Acetamide^b^	0.19 ± 0.06	0.06 ± 0.01	0.06 ± 0.01	0.06 ± 0.01	0.11 ± 0.01	0.06 ± 0.01	0.05 ± 0.01	0.05 ± 0.01

a,b *Refers to the different configurations of the same substance; ND, no data were obtained*.

### Metabolomic Analysis by GC–MS

A total of 101 metabolites including 21 amino acids, 18 sugars, 6 alcohols, and their derivatives were detected and quantified by GC–MS. The data discussed in this section are shown in Table 1, and the relative contents of other intercellular metabolites are shown in Supplementary Table 1.

As shown in Table 1, after 48-h fermentation, the contents of amino acids, especially for the ε-PL precursors such as asparagine (Asn), aspartic acid (Asp), and lysine (Lys), in the high-yield strain 6#-7 were larger than those in the original strain TUST, indicating that the high-yield strain can produce more products in key steps of the ε-PL synthesis pathway. Among the 18 sugars, the glucose content in strain 6#-7 was significantly lower than that in strain TUST, indicating that 6#-7 has a stronger glucose-consumption ability, which is conducive to the production of ε-PL yield (Pan et al., 2019). Also, maltose content in the branched-chain ε-PL metabolic pathway was lower in 6#-7, which benefits the accumulation of ε-PL. On the contrary, increased trehalose during the initial growth stage of 6#-7 would enhance its resistance to ε-PL (Sanchez et al., 2010). The contents of 28 organic acids, hexadecenoic acid, octadecanoic acid, and other fatty acids in strain 6#-7 were significantly lower than those in strain TUST, indicating that the fatty acid branch metabolism pathway in 6#-7 is weakened and thus the consumption of acetyl-CoA (Accoa) is reduced. Also, all of these benefit the accumulation of ε-PL in 6#-7. The contents of other compounds, such as norvaline, sedoheptulose, dodecanol, and acetamide, did not show any obvious difference between the two strains.

### Principal Component Analysis (PCA) and Partial Least Squares Analysis (PLSA)

Unsupervised (i.e., PCA) and supervised (i.e., PLSA) approaches were used to classify the data set of different strains. The values of *R*^2^ and *Q*^2^ represent the accuracy of fit and the predictability of the model, respectively. It is considered that if *R*^2^ and *Q*^2^ are greater than 0.9, the model is valid, and generally, it is reasonable if the values are greater than 0.5. The main statistics are shown in Supplementary Table 2. It shows that the values of *R*^2^ and *Q*^2^ are close to 1.0 except for the values of *Q*^2^ of PCA, indicating that the model is credible.

The scores and loadings represent the discrete (Jackson, 1980) and central tendency of the data (Kettaneh et al., 2005). Specifically, discrete tendency reflects the differences among the variables, while central tendency reflects the similarity among the variables (Wold et al., 1987).

**Figure 2:**
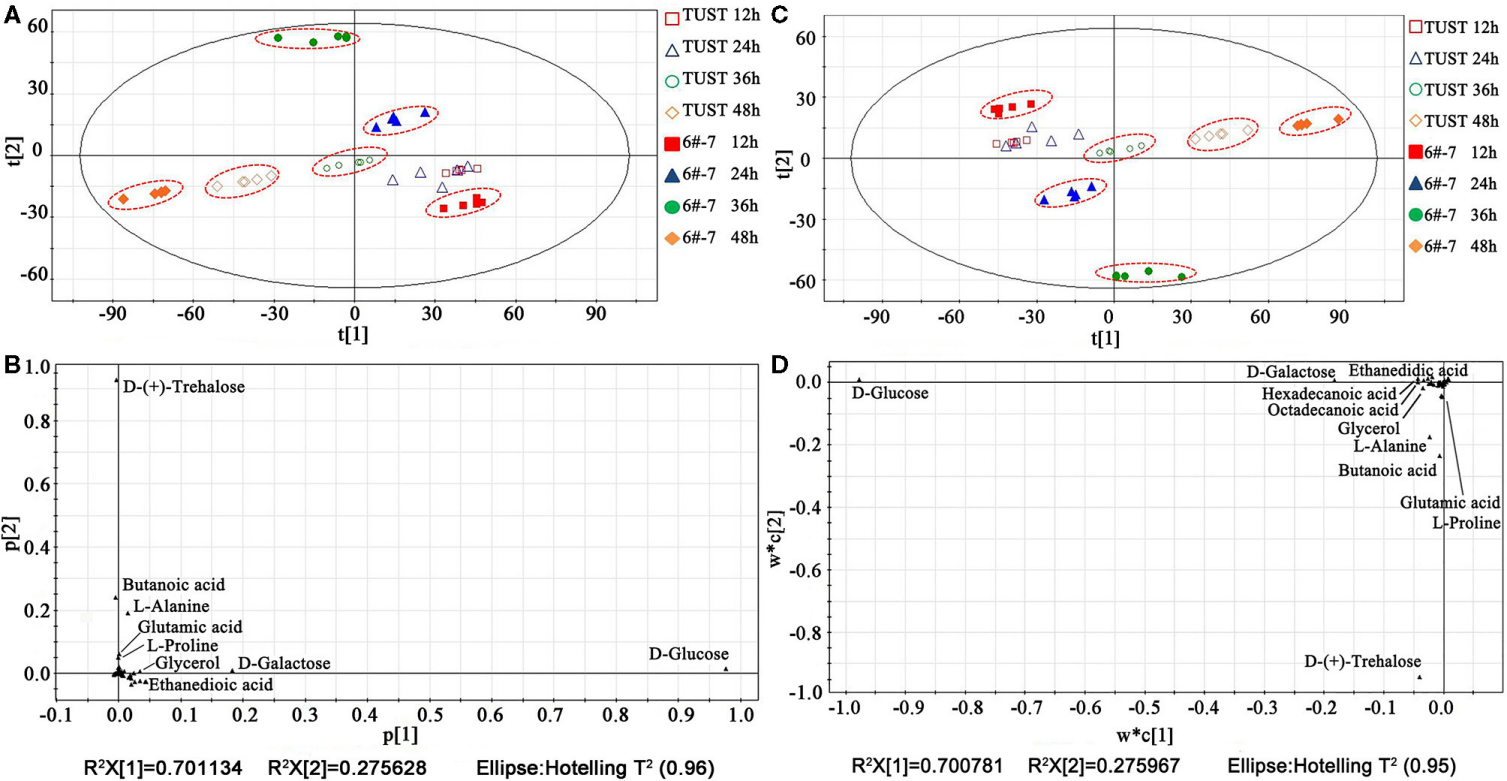
Principle component analysis (PCA) and partial least squares analysis (PLSA) of the metabolites at different fermentation stages: score plot **(A)** and loading plot **(B)** by PCA; score plot **(C)** and loading plot **(D)** by PLSA.

Each experiment was repeated five times to ensure the reproducibility of the results, and the data were initially classified by the PCA. As shown in Figure 2A, the data of the same group were centralized, indicating that the model has good testing repeatability. The distribution of each group, except for TUST at 12 and 24 h, was discrete from other groups, meaning there were significant differences in the metabolisms between TUST and 6#-7 during fermentation periods. The data of TUST at 12 h and 24 h were clustered, indicating that the metabolism was stable for TUST at the initial stage. The loading plot was used to find biomarkers. The farther a metabolite is away from the origin of coordinates, the greater contribution it makes (Teague et al., 2007). As shown in Figure 2B, the metabolites with a high contribution rate during the whole fermentation process were d-(+)-trehalose, d-glucose (d*-*Glc), d-galactose, butanoic acid, l-alanine (l*-*Ala), oxalic acid, glutamic acid (Glu), glycerol, and l-valine.

To further confirm the results of PCA, the PLSA was also used for the statistical analysis (Wang, 1999). As shown in Figure 2C, the results were consistent with those of the PCA, indicating that the metabolisms of TUST and 6#-7 were significantly different during fermentation periods. As shown in Figure 2D, there were other major metabolites, such as palmitic acid and stearic acid, besides those found in the PCA.

Under the PLSA model, a multidimensional analysis of differential metabolite screening can be performed by the VIP analysis. The VIP coefficients reflect the contribution of each metabolite to the PLSA models; a higher VIP value indicates that the metabolite has a larger contribution (Lv et al., 2016). In this study, a VIP value of 0.5 was used as the cutoff value for statistical significance, and thus more potential biomarkers can be screened.

As shown in Supplementary Figure 2, biomarkers extracted from the loading plot can be sorted according to the contribution rate as follows: d*-*Glc > d-galactose > d-(+)-trehalose > stearic acid > palmitic acid > glycerol > l*-*Ala and oxalic acid. Among them, d*-*Glc, d-galactose, and d-(+)-trehalose are the main intermediates related to sugar metabolism. Stearic acid and palmitic acid are the important products of the fatty acid metabolism pathway. l*-*Ala and oxalic acid are closely related to cell center carbon metabolism.

Hierarchical cluster analysis (HCA) was performed to further validate the prediction accuracy of the PCA model. Heatmap generated from HCA based on the first principal component is shown in Supplementary Figure 3. The samples have obvious distinctions and can be sorted according to the strains, indicating that the HCA results are consistent with those of PCA.

### Comparative Analysis of Intracellular Metabolites Related to ε-PL Synthesis

**Figure 3:**
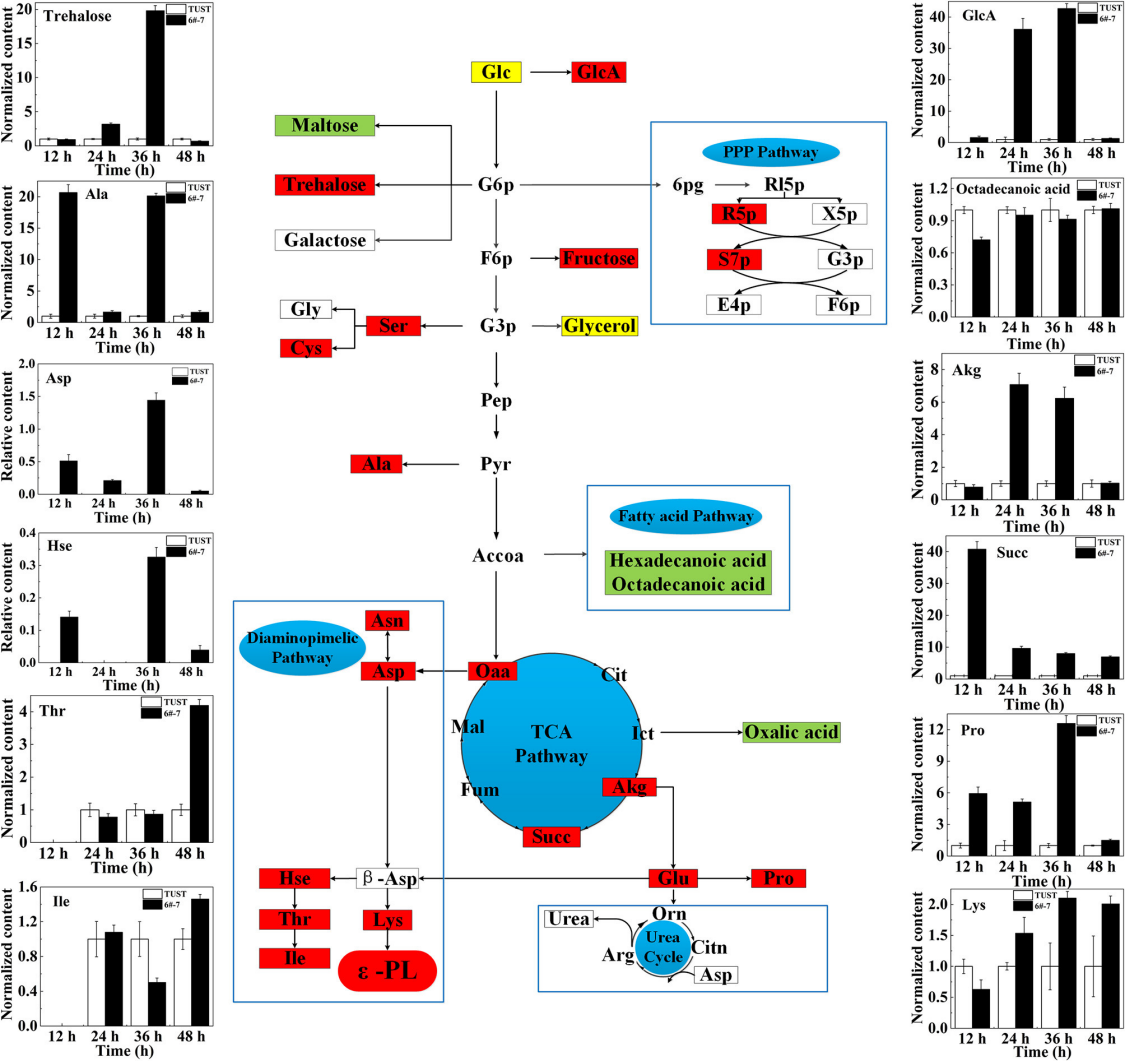
Schematic diagram showing the changes of differential metabolite content mapped onto the metabolic network in strain 6#-7. The flow diagram just reflects the trends when the differences between 6#-7 and the original strain were the biggest. Red ones: increase in content; green ones: decrease in content; and yellow ones: first increased and then decreased. Glc, glucose; GlcA, gluconic acid; G6p, glucose-6-phosphate; 6pg, 6-phosphate gluconic acid; Rl5p, ribulose-5-phosphate; R5p, ribose-5-phosphate; X5p, xylulose-5-phosphate; S7p, sedoheptulose-7-phosphate; G3p, glyceraldehyde-3-phosphate; E4p, erythrose-4-phosphate; F6p, fructose-6-phosphate; Gly, glycine; Ser, serine; Cys, cystathionine; Pep, phosphoenolpyruvic acid; Pyr, pyruvate; Ala, alanine; Accoa, acetyl-CoA; Oaa, oxaloacetate; Cit, citrate; Ict, isocitrate; Akg, α-ketoglutarate; Succ, succinate; Fum, fumarate; Mal, malic acid; Asn, asparagine; Asp, aspartic acid; β-Asp, β-aspartic acid; Hse, homoserine; Thr, threonine; Ile, isoleucine; Lys, lysine; Glu, glutamic acid; Pro, proline; Orn, ornithine; Citn, citrulline; Arg, arginine. Except for the contents of Asp and Hse, which were undetectable in the original strain, the content of all the other metabolites in 6#-7 was normalized by that of the original strain.

The normalized relative contents of differential intracellular metabolites between TUST and 6#-7 are shown in Figure 3. It shows that strain 6#-7 has a strong ability to absorb glucose. Also, the weakening of the fatty acid metabolism pathway liberates more Accoa to the tricarboxylic acid cycle (TCA), which can promote the TCA pathway and increase the accumulation of Oaa. Then, the pathway from Oaa to l*-*Lys is enhanced. This provides sufficient energy for the synthesis of Oaa and precursor l*-*Lys, which ultimately leads to higher ε-PL productivity (Zhang et al., 2018).

The enhancement of the diaminopimelate pathway increases the content of threonine (Thr), homoserine (Hse), and isoleucine (Ile), and that promotes the accumulation of Lys, which is the precursor of ε-PL (Xiang et al., 2020). In addition, it is presumed that there may be a pathway that can compensate for the loss of intermediate metabolites in the pentose phosphate pathway (PPP) and allow the PPP to be restored, which in turn provides a large amount of NADPH for ε-PL synthesis. Moreover, the increase of α-ketoglutarate (Akg) and succinic acid (Succ) promotes the production of proline (Pro) and Glu, which enhances the ability of the strain itself to resist ε-PL (Brandriss and Falvey, 1992; Guan et al., 2013). Thus, the ability of the mutagenized strain to synthesize ε-PL is enhanced.

**Figure 4:**
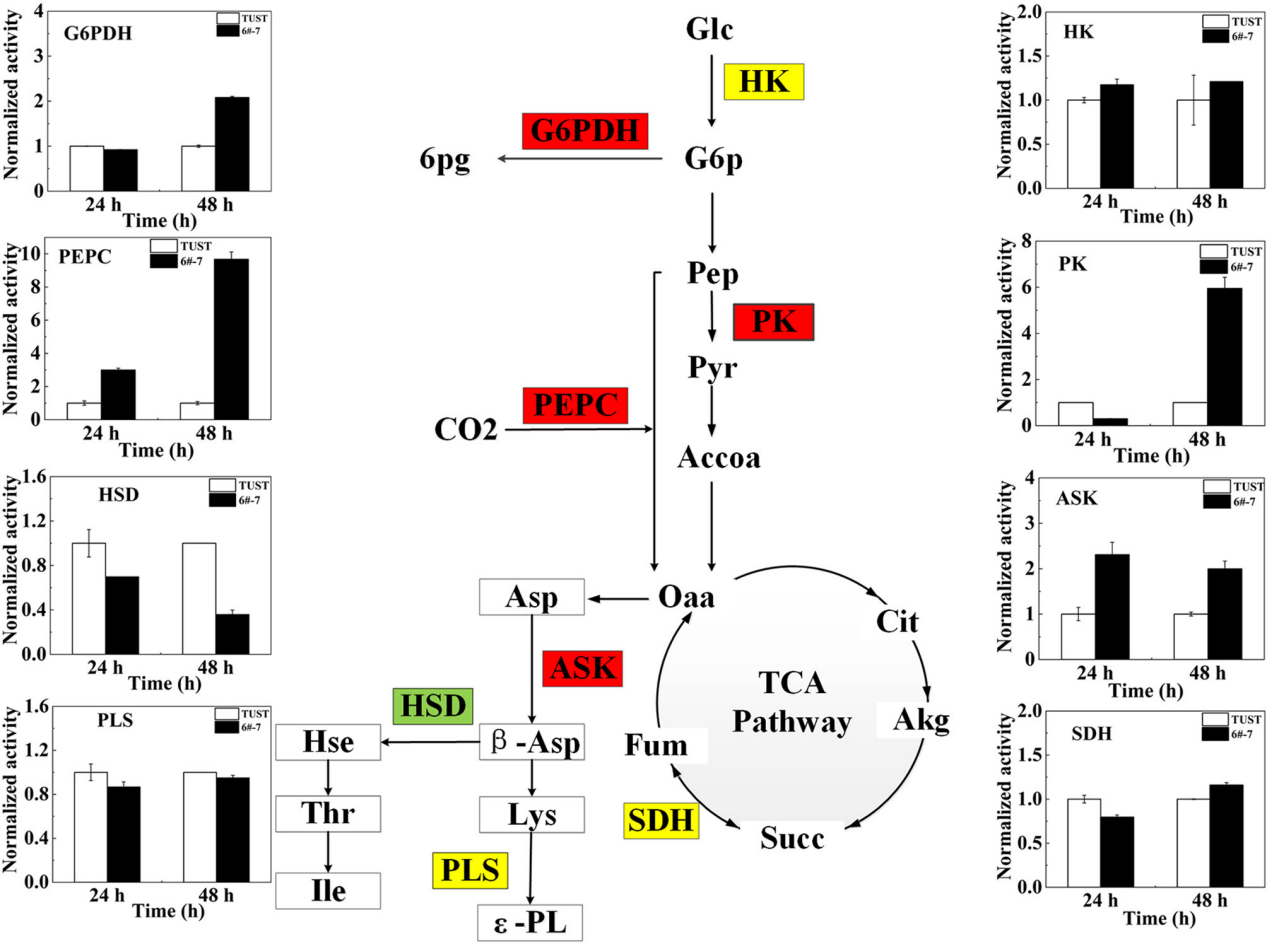
Changes of key enzyme activity in the strain before and after mutagenesis. The flow diagram just reflects the trends when the differences between 6#-7 and the original strain were the biggest. Red ones: increase in activity; green ones: decrease in activity; and yellow ones: no obvious change. HK, hexokinase; PK, pyruvate kinase; PEPC, phosphoenolpyruvate carboxylase; ASK, aspartokinase; HSD, homoserine dehydrogenase; G6PDH, glucose-6-phosphate dehydrogenase; SDH, succinate dehydrogenase; PLS, polylysine synthetase.

In general, based on the metabolomic analysis, it was found that the intracellular metabolism of the strain significantly changed after mutagenesis, which is consistent with the results of PCA and PLSA. Moreover, combined with Figure 1, it is indicated that the difference in metabolism between the two strains occurred earlier. Also, except for the normalized contents of Thr, Ile, and Lys that showed the biggest difference between TUST and 6#-7 at 48 h, the contents of all the other 9 metabolites showed the biggest difference at 12, 24, or 36 h. This is because of Thr, Ile, and Lys which are very close to ε-PL in the metabolic network, and they showed the biggest difference when the strain began to produce ε-PL in large quantities (Figures 1, 4). On the contrary, the other metabolites are far away from or not that close to ε-PL in the metabolic network, and they showed the biggest difference mainly during the growth stage of the strain.

Based on the findings of main metabolites by the abovementioned loading plot analysis and the analysis of related metabolic intermediates in the metabolite network, a possible metabolic regulation mechanism of the high-yield mutagenized strain was revealed. First, the transport and absorption capacity for Glc of strain 6#-7 is improved. Second, the TCA pathway is promoted and that can increase the accumulation of the precursor Oaa. Third, the diaminopimelate pathway is enhanced to produce more Lys, which is an essential precursor of ε-PL synthesis. Finally, the increase of trehalose, Glu, and Pro makes the strain 6#-7 more resistant to ε-PL.

### Comparative Analysis of Key Enzyme Activities in Metabolic Pathways During ε-PL Synthesis

The enzyme plays an important role in the growth and metabolism of microorganisms (Teague et al., 2007); thus, the activities of enzyme in metabolic pathways associated with ε-PL synthesis were measured to gain further insight into the biosynthesis mechanism of ε-PL in the high-yield strain 6#-7 at the enzymatic level.

The activities and normalized activities of the key enzymes are shown in Supplementary Figure 4 and Figure 4, respectively. During the metabolic synthesis in microorganisms, the PPP mainly provides NADPH (Kind et al., 2013). Increased G6PDH activity indicates the enhancement of the PPP that can provide a large amount of NADPH for the synthesis of ε-PL (Hamano, 2010). The activity of G6PDH increased in strain 6#-7 benefits the production of ε-PL. The activity of PK in the high-yield strain 6#-7 is strengthened, indicating that the glycolysis pathway (EMP) pathway in strain 6#-7 is still unblocked which can provide sufficient pyruvate (Pyr) (Calmettes et al., 2015; Alves-Filho and PåLsson-Mcdermott, 2016).

The increase in the activity of PEPC in strain 6#-7 benefits the accumulation of Oaa (Atsushi and Shiio, 2016). Besides, the increasing activity of PEPC can also provide more NADPH for ε-PL synthesis. Also, the enhancement of ASK activity can provide more β-aspartic acid (β-Asp) in strain 6#-7 for the synthesis of Lys (Wittmann and Becker, 2007), which is the precursor of ε-PL. Meanwhile, the decrease in the activity of HSD leads to less Hse to the branched-chain pathways in strain 6#-7 (Hamano, 2010). All of these promote the production of ε-PL in the high-yield strain 6#-7. In addition, the activities of HK, SDH, and PLS change a few in strain 6#-7 as compared with the original strain.

### Metabolomic and Enzymatic Analyses in ε-PL Synthesis

**Figure 5:**
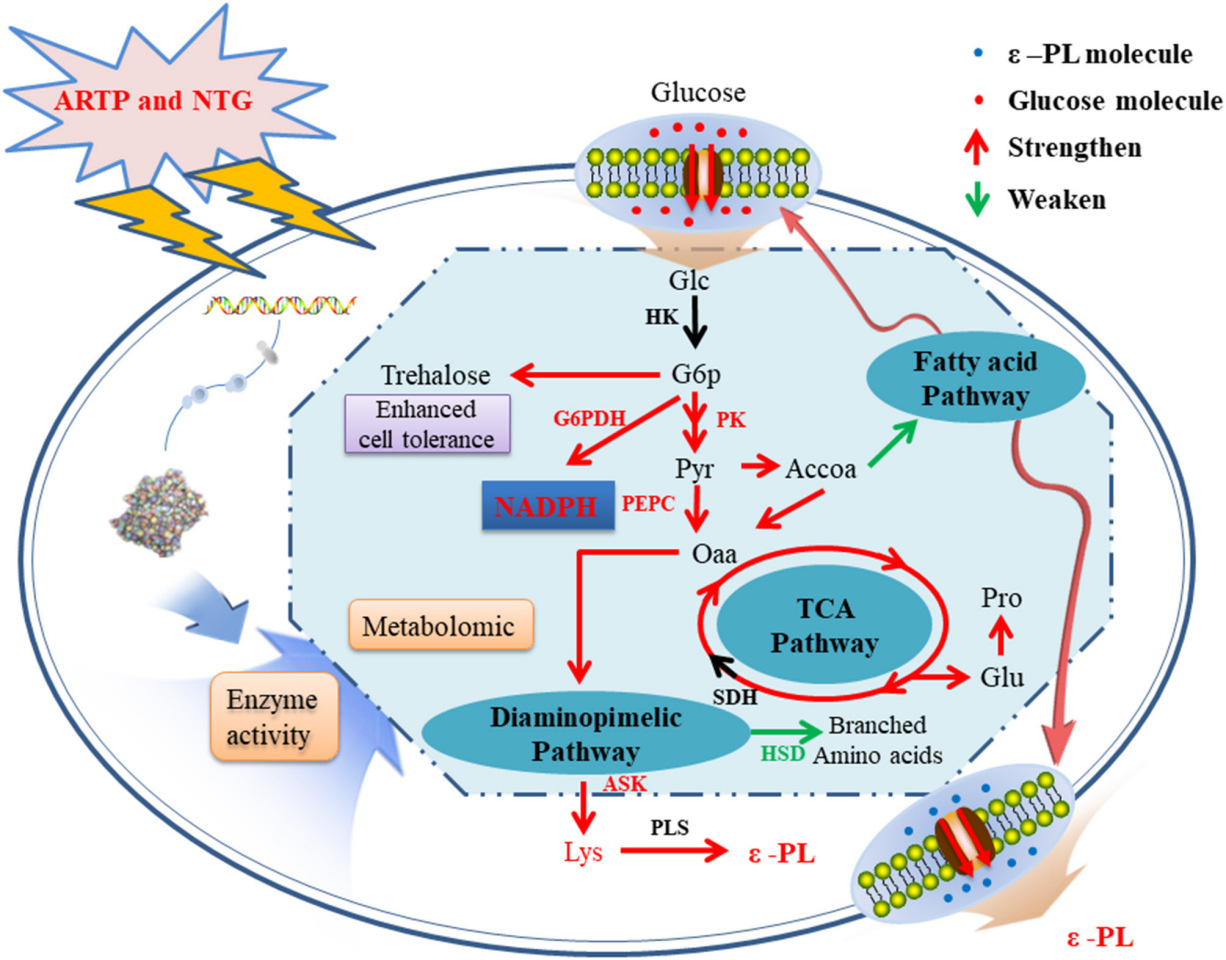
A possible metabolic regulation mechanism of the high-yield ε-PL mutagenized strains: the transport and absorption capacity for glucose of strain 6#-7 is improved; the activity of enzymes benefits ε-PL synthesis is strengthened; on the contrary, the activity of enzyme in the branched-chain pathways is decreased; besides, the increase of trehalose, glutamic acid, and proline makes the strain 6#-7 more resistant to ε-PL.

Based on the results of metabolomic and enzymatic analyses, a possible metabolic regulation mechanism of the high-yield mutagenized strain was revealed (Figure 5). First, the transport and absorption capacity for Glc of strain 6#-7 is improved. Second, the activity of enzymes benefits ε-PL synthesis, such as G6PDH, PK, PEPC, and ASK, is strengthened. On the contrary, the activity of HSD in the branched-chain pathways is decreased. Third, the increase of trehalose, Glu, and Pro makes the strain 6#-7 more resistant to ε-PL. Thus, the ability of the mutagenized strain to synthesize ε-PL is enhanced, and strain 6#-7 can produce more ε-PL compared with the original strain. To analyze a mutant strain, genomic sequencing is also a useful method (Wang et al., 2015; Hwang et al., 2019). Thus, genomic sequencing was conducted in another study by our laboratory that further improve the production of ε-PL using genetic engineering (i.e., data were not shown in this study). Also, the results obtained by the genetic analysis are highly consistent with those obtained by metabolomic and enzymatic analyses in this study.

## Conclusion

In this study, the biosynthesis mechanism of ε-PL produced by the high-yield strain 6#-7 was studied. It was found that the metabolism map of the high-yield strain changed significantly compared with the origin strain. The scores of the PCA and PLSA analyses showed that there were significant differences in intracellular metabolism during different periods of fermentation. The main metabolites that cause cell variability by loading plot analysis may be d*-*Glc, d-galactose, d-(+)-trehalose, stearic acid, palmitic acid, glycerol, l*-*Ala, and oxalic acid. Unlike the origin strain, the ability of strain 6#-7 cells to transport and absorb glucose is improved. The EMP and TCA pathways are activated earlier, and the diaminopimelate pathway is enhanced by providing a large amount of ε-PL precursor such as Lys. Moreover, the increased protective substances such as trehalose, Glu, and Pro enhance the ability of strain 6#-7 itself to resist ε-PL.

The activities of key enzymes involved in the metabolism pathway related to ε-PL synthesis were also examined, and the reasons for the differences between the two strains in the ability to synthesize ε-PL were explained at the enzymatic level. The increase of G6PDH, PK, PEPC, and ASK activities together with the reduction of HSD activity promote the production of ε-PL. This is the first study in which the metabolomic analysis of the biosynthesis mechanism of ε-PL that was produced by the high-yield strain 6#-7 was systematically studied. These findings provide a theoretical basis for further improving the production of ε-PL.

## Data Availability Statement

The original contributions presented in the study are included in the article/Supplementary Material, further inquiries can be directed to the corresponding authors.

## Author Contributions

JC, SJ, and ZiyW designed the study. FG and TD performed the experiments. ZiyW, FG, TD, and SJ analyzed the data. ZiyW, TD, ZT, MA, ZicW, and JC wrote or contributed to the writing of the manuscript. All authors contributed to the article and approved the submitted version.

## Conflict of Interest

ZiyW was employed by company Tianjin Beiyang Baichuan Biotechnology Co., Ltd. The remaining authors declare that the research was conducted in the absence of any commercial or financial relationships that could be construed as a potential conflict of interest.

## Publisher's Note

All claims expressed in this article are solely those of the authors and do not necessarily represent those of their affiliated organizations, or those of the publisher, the editors and the reviewers. Any product that may be evaluated in this article, or claim that may be made by its manufacturer, is not guaranteed or endorsed by the publisher.
