# Supplementary material for: Metabolomic Analysis of Biosynthesis Mechanism of ε-Polylysine Produced by Streptomyces diastatochromogenes
Source: Front Bioeng Biotechnol. 2021 Jul 30;9:698022. doi: 10.3389/fbioe.2021.698022 (PMC8363252; doi:10.3389/fbioe.2021.698022)
Supplement: Supplementary file 1 [file Data_Sheet_1.docx]

Supplementary Material

**TABLE S1****.** Relative intracellular metabolite contents identified by *GC-MS*

| Metabolites | TUST | | | | 6#-7 | | | |
| --- | --- | --- | --- | --- | --- | --- | --- | --- |
|  | 12 h | 24 h | 36 h | 48 h | 12 h | 24 h | 36 h | 48 h |
| Amino acid | | | | | | | | |
| Valine^1^ | ND | ND | 0.31±0.01 | 0.15±0.04 | ND | 0.11±0.0 | 0.25±0.03 | 0.53±0.02 |
| Alanine^1^ | 0.60±0.19 | 0.46±0.14 | 1.08±0.11 | 0.95±0.21 | 12.37±1.35 | 0.76±0.10 | 21.78±0.46 | 1.39±0.25 |
| Leucine^1^ | ND | ND | 0.65±0.13 | 0.37±0.06 | ND | ND | 0.06±0.01 | 1.07±0.04 |
| Isoleucine^1^ | ND | ND | 0.06±0.01 | 0.09±0.01 | ND | 0.02±0.0 | 0.07±0.01 | 0.26±0.02 |
| Glycine^1^ | 0.15±0.01 | 0.09±0.01 | 0.09±0.01 | 0.09±0.01 | 0.13±0.01 | 0.09±0.01 | 0.08±0.01 | 0.09±0.01 |
| Valine^2^ | ND | 0.19±0.04 | 0.52±0.09 | 0.45±0.06 | 0.15±0.07 | 0.19±0.02 | 0.36±0.02 | 0.65±0.04 |
| Cystathionine^1^ | ND | ND | ND | 0.03±0.01 | 0.04±0.01 | 0.03±0.01 | 0.03±0.01 | 0.08±0.01 |
| Cystathionine^2^ | ND | ND | ND | ND | ND | 0.02±0.01 | 0.03±0.01 | ND |
| Serine^1^ | ND | ND | ND | ND | ND | ND | 0.14±0.01 | 0.22±0.02 |
| Leucine^2^ | ND | 0.25±0.05 | 0.74±0.18 | 0.75±0.15 | ND | ND | 0.04±0.01 | 0.89±0.08 |
| Isoleucine^2^ | ND | 0.09±0.02 | 0.22±0.05 | 0.23±0.04 | ND | 0.07±0.01 | 0.07±0.01 | 0.2±0.02 |
| Threonine^1^ | ND | ND | ND | ND | ND | ND | ND | 0.37±0.04 |
| Homoserine | ND | ND | ND | ND | 0.14±0.02 | ND | 0.33±0.03 | 0.03±0.01 |
| Serine^2^ | ND | 0.07±0.01 | 0.09±0.01 | 0.09±0.01 | ND | 0.07±0.01 | 0.10±0.04 | 0.14±0.01 |
| Threonine^2^ | ND | 0.10±0.02 | 0.15±0.03 | 0.14±0.02 | ND | 0.08±0.01 | 0.13±0.01 | 0.21±0.02 |
| Methionine^1^ | ND | ND | ND | ND | ND | ND | ND | 0.04±0.01 |
| Glycine^2^ | 2.03±0.03 | 1.18±0.12 | 1.18±0.04 | 1.09±0.02 | 1.71±0.04 | 1.25±0.05 | 1.18±0.01 | 1.16±0.08 |
| N-α-Acetyl-Lysine | 2.97±0.90 | 1.64±0.28 | 2.05±0.12 | 1.86±0.21 | 2.62±0.28 | 1.86±0.07 | 1.62±0.07 | 1.58±0.13 |
| Methionine^2^ | ND | ND | 0.03±0.01 | 0.04±0.01 | ND | ND | ND | 0.07±0.01 |
| Proline^1^ | ND | ND | 0.06±0.02 | 0.04±0.01 | ND | ND | ND | 0.04±0.01 |
| Proline^2^ | 0.13±0.03 | 0.37±0.17 | 0.26±0.04 | 0.21±0.01 | 0.79±0.08 | 1.91±0.11 | 4.15±0.25 | 0.35±0.03 |
| Glycyl-l-glutamic acid | ND | ND | ND | ND | 1.74±0.27 | 0.66±0.12 | 1.14±0.13 | ND |
| Alanine^2^ | ND | ND | ND | ND | ND | ND | ND | 0.13±0.03 |
| Glutamic acid | ND | 0.04±0.01 | 0.12±0.03 | 0.12±0.03 | 1.58±0.46 | 1.62±0.20 | 5.31±0.24 | 0.16±0.02 |
| Phenylalanine | ND | 0.04±0.01 | 0.13±0.02 | 0.16±0.03 | 0.04±0.01 | 0.02±0.01 | 0.03±0.01 | 0.24±0.03 |
| Ornithine | ND | 0.01±0.01 | 0.02±0.01 | 0.01±0.01 | ND | 0.01±0.01 | 0.12±0.01 | 0.04±0.01 |
| Tyrosine | 0.09±0.01 | 0.05±0.02 | 0.16±0.03 | 0.08±0.02 | 0.06±0.01 | 0.05±0.01 | 0.05±0.01 | 0.13±0.03 |
| Carbohydrate | | | | | | | | |
| α-Glucopyranoside^1^ | 0.06±0.01 | 0.04±0.01 | 0.02±0.01 | 0.04±0.01 | 0.07±0.01 | 0.04±0.01 | 0.07±0.01 | 0.05±0.01 |
| α-Glucopyranoside^2^ | 0.12±0.02 | 0.07±0.01 | 0.07±0.01 | 0.06±0.01 | 0.08±0.01 | 0.07±0.01 | 0.06±0.01 | 0.07±0.01 |
| β-Galactopyranoside^1^ | ND | ND | ND | ND | ND | 0.02±0.01 | 0.06±0.01 | 0.02±0.01 |
| Ribose^1^ | 0.12±0.01 | 0.07±0.02 | 0.09±0.01 | 0.06±0.01 | 0.11±0.02 | 0.11±0.01 | 0.25±0.01 | 0.06±0.01 |
| Arabinofuranose^1^ | ND | 0.01±0.01 | 0.01±0.01 | 0.04±0.01 | 0.01±0.01 | 0.03±0.01 | 0.08±0.01 | 0.02±0.01 |
| α-Galactopyranose | ND | 0.18±0.01 | 0.06±0.01 | 0.07±0.01 | ND | 0.36±0.03 | 0.42±0.01 | 0.07±0.01 |
| β-Galactofuranose | ND | 0.02±0.01 | ND | ND | ND | 0.01±0.01 | ND | ND |
| Fructose | ND | 0.03±0.01 | ND | ND | ND | 0.03±0.01 | 0.08±0.01 | 0.02±0.01 |
| Galactose^1^ | ND | 0.04±0.01 | 0.08±0.01 | 0.2±0.04 | 0.02±0.01 | 0.02±0.01 | 0.21±0.06 | 1.05±0.04 |
| Glucopyranose^1^ | 1.24±0.17 | 1.01±0.21 | 0.23±0.05 | 0.04±0.01 | 0.78±0.06 | 0.61±0.14 | 1.04±0.05 | ND |
| Galactose^2^ | 8.63±0.16 | 21.80±1.73 | 1.94±0.31 | 0.62±0.14 | 27.95±0.80 | 14.61±0.55 | 12.7±0.14 | 0.04±0.01 |
| Arabinofuranose^2^ | 0.01±0.01 | 0.03±0.01 | 0.01±0.01 | 0.01±0.01 | 0.05±0.01 | 0.05±0.01 | 0.05±0.01 | ND |
| Glucopyranose^2^ | 2.42±0.14 | 3.03±0.91 | 0.39±0.02 | 0.14±0.04 | 2.75±0.11 | 1.69±0.17 | 1.61±0.13 | ND |
| Xylopyranose | 0.12±0.04 | 0.46±0.15 | 0.03±0.01 | 0.04±0.01 | 0.45±0.06 | 0.24±0.03 | 0.26±0.02 | ND |
| Ribose^2^ | ND | ND | 0.02±0.01 | 0.02±0.01 | 0.02±0.01 | 0.02±0.01 | 0.03±0.01 | 0.02±0.01 |
| Melibiose | ND | 0.05±0.02 | 0.05±0.02 | 0.04±0.01 | 0.09±0.01 | 0.15±0.02 | 0.48±0.02 | 0.1±0.01 |
| Mannose^1^ | ND | ND | ND | ND | ND | 0.05±0.01 | 0.17±0.04 | ND |
| Mannose^2^ | ND | 0.04±0.02 | ND | ND | 0.02±0.01 | 0.16±0.02 | 0.59±0.04 | 0.01±0.01 |
| Erythro-Pentose | 2.12±0.21 | 1.21±0.40 | 1.05±0.08 | 0.99±0.12 | 1.25±0.19 | 0.95±0.11 | 1.01±0.03 | 1.01±0.10 |
| β-Galactopyranoside^2^ | 7.78±0.30 | 6.89±0.46 | 3.37±1.33 | 3.58±1.36 | 4.60±1.06 | 2.63±0.60 | 5.27±0.39 | 4.14±1.11 |
| Altro-2-Heptulose | ND | ND | ND | ND | ND | ND | 0.08±0.01 | 0.01±0.01 |
| Alcohol | | | | | | | | |
| Glycerol | 3.10±0.90 | 2.68±0.52 | 2.78±0.28 | 2.42±0.20 | 6.05±0.33 | 5.64±0.17 | 2.74±0.04 | ND |
| Xylitol | 0.04±0.01 | 0.04±0.01 | 0.04±0.01 | 0.02±0.01 | 0.04±0.01 | 0.04±0.01 | ND | ND |
| β-Eudesmol | ND | ND | ND | ND | ND | ND | 0.05±0.01 | 0.04±0.01 |
| Inositol^1^ | 0.03±0.01 | 0.02±0.01 | 0.03±0.01 | 0.04±0.01 | 0.03±0.01 | 0.02±0.01 | 0.04±0.01 | 0.03±0.01 |
| Inositol^2^ | 0.16±0.02 | 0.05±0.02 | 0.07±0.01 | 0.05±0.01 | 0.07±0.01 | 0.06±0.01 | 0.12±0.02 | 0.07±0.01 |
| Myo-Inositol | ND | 0.03±0.01 | 0.06±0.01 | 0.03±0.01 | 0.01±0.01 | 0.03±0.01 | 0.06±0.01 | 0.06±0.01 |
| Organic acid | | | | | | | | |
| Lactic acid | 0.19±0.05 | 0.13±0.05 | 0.14±0.04 | 0.13±0.03 | 0.15±0.03 | 0.17±0.01 | 0.12±0.01 | 0.15±0.02 |
| Propanoic acid^1^ | ND | 0.04±0.01 | 0.04±0.01 | 0.05±0.01 | 0.05±0.01 | 0.04±0.01 | 0.06±0.01 | 0.03±0.01 |
| Propanoic acid^2^ | 4.10±0.33 | 3.47±0.40 | 3.11±0.44 | 2.59±0.35 | 3.63±0.16 | 3.19±0.15 | 1.82±0.16 | 1.94±0.14 |
| Hexanoic acid^1^ | 0.02±0.01 | 0.17±0.01 | 0.02±0.01 | 0.01±0.01 | 0.03±0.01 | 0.01±0.01 | 0.02±0.01 | 0.01±0.01 |
| Acetic acid | 0.03±0.01 | 0.01±0.01 | ND | ND | 0.03±0.01 | 0.01±0.01 | ND | 0.01±0.01 |
| Ethanedioic acid^1^ | 9.06±1.30 | 5.29±0.68 | 6.61±1.07 | 5.26±0.59 | 5.78±0.48 | 5.01±0.55 | 2.83±0.26 | 4.56±0.41 |
| 4-Octenoic acid | 0.14±0.02 | 0.08±0.01 | 0.1±0.01 | 0.08±0.01 | 0.13±0.02 | 0.18±0.01 | 0.12±0.05 | 0.07±0.01 |
| Benzoic acid | ND | ND | ND | ND | 0.13±0.02 | 0.04±0.01 | 0.10±0.01 | 0.03±0.01 |
| 2-Piperidinecarboxylic acid | ND | ND | ND | ND | ND | 0.07±0.01 | 0.13±0.01 | ND |
| 1-Aminocyclopentanecar  Boxylic acid | ND | 0.07±0.01 | 0.06±0.01 | 0.04±0.01 | ND | 0.06±0.01 | 0.34±0.11 | 0.06±0.01 |
| 1-Methylcyclohexanecar  Boxylic acid | 0.06±0.01 | 0.03±0.01 | 0.04±0.01 | 0.03±0.01 | 0.05±0.01 | 0.04±0.01 | 0.04±0.00 | 0.04±0.01 |
| Ethanedioic acid^2^ | 21.28±0.28 | 20.02±0.76 | 19.56±0.02 | 20.00±0.27 | 26.52±0.07 | 20.23±0.02 | 18.70±0.14 | 18.77±0.17 |
| Butanedioic acid^1^ | 0.03±0.01 | 0.11±0.01 | 0.27±0.04 | 0.08±0.01 | 1.25±0.07 | 1.17±0.09 | 1.97±0.08 | 0.53±0.03 |
| Hexanoic acid^2^ | ND | ND | ND | 0.01±0.01 | 0.01±0.01 | 0.01±0.01 | 0.06±0.02 | 0.02±0.01 |
| Benzenepropanoic acid | 0.03±0.01 | 0.02±0.01 | 0.02±0.01 | 0.02±0.01 | 0.02±0.01 | 0.03±0.01 | 0.06±0.01 | 0.03±0.01 |
| Nonanoic acid | 0.13±0.02 | 0.07±0.01 | 0.06±0.01 | 0.05±0.01 | 0.09±0.01 | 0.06±0.01 | 0.06±0.01 | 0.05±0.01 |
| Butanedioic acid^2^ | ND | 0.03±0.01 | 0.01±0.01 | 0.01±0.01 | ND | 0.12±0.01 | 0.22±0.01 | 0.01±0.01 |
| Butanoic acid^1^ | 0.11±0.02 | 0.06±0.01 | 0.10±0.01 | 0.07±0.01 | 0.08±0.01 | 0.06±0.01 | 0.07±0.01 | 0.07±0.01 |
| Isophthalic acid | ND | ND | ND | ND | ND | ND | 0.18±0.01 | ND |
| Butanoic acid^2^ | 0.28±0.04 | 0.13±0.02 | 0.16±0.03 | 0.14±0.02 | 0.23±0.01 | 0.17±0.01 | 0.16±0.03 | 0.15±0.01 |
| 2,3,4-Trimethyl-3-  Hydroxyglutaric acid | ND | 0.04±0.01 | 0.01±0.01 | 0.04±0.01 | ND | ND | ND | ND |
| Pentanedioic acid | 0.08±0.01 | 0.01±0.01 | 0.04±0.01 | 0.04±0.01 | 0.07±0.01 | 0.05±0.01 | 0.24±0.03 | 0.05±0.01 |
| Dodecanoic acid | 0.13±0.02 | 0.07±0.01 | 0.08±0.01 | 0.09±0.01 | 0.1±0.01 | 0.08±0.01 | 0.08±0.01 | 0.08±0.01 |
| Tetradecanoic acid^1^ | ND | 0.02±0.0 | 0.02±0.0 | 0.02±0.0 | ND | ND | 0.02±0.0 | 0.02±0.0 |
| Propanoic acid^4^ | ND | ND | ND | ND | ND | 0.01±0.01 | 0.14±0.04 | ND |
| Tetradecanoic acid^2^ | 0.30±0.03 | 0.19±0.07 | 0.20±0.02 | 0.17±0.01 | 0.31±0.02 | 0.16±0.01 | 0.16±0.01 | 0.17±0.01 |
| Phthalic acid^1^ | 0.07±0.01 | 0.07±0.02 | 0.04±0.01 | 0.04±0.01 | 0.09±0.01 | 0.04±0.01 | 0.07±0.02 | 0.02±0.01 |
| n-Pentadecanoic acid^1^ | ND | ND | 0.01±0.01 | 0.04±0.01 | ND | ND | 0.15±0.03 | 0.04±0.01 |
| N,N,O-Tris-(trimethylsilyl)-  4-aminomethy-  Lcyclohexane  Carboxylic acid | ND | ND | ND | ND | 0.06±0.01 | 0.05±0.01 | 0.06±0.01 | 0.15±0.02 |
| n-Pentadecanoic acid^2^ | ND | ND | ND | ND | 0.01±0.01 | 0.01±0.01 | 0.01±0.01 | 0.04±0.01 |
| Phthalic acid^2^ | 0.08±0.01 | 0.06±0.01 | 0.04±0.01 | 0.04±0.01 | 0.08±0.02 |  | 0.03±0.01 | 0.02±0.01 |
| Decanoic acid | 0.05±0.01 | 0.04±0.01 | 0.03±0.01 | 0.03±0.01 | 0.03±0.01 | 0.03±0.01 | 0.03±0.01 | 0.02±0.01 |
| Gluconic acid | 0.01±0.01 | 0.03±0.01 | 0.01±0.01 | 0.01±0.01 | 0.02±0.01 | 0.02±0.01 | 0.09±0.01 | ND |
| Heptadecanoic acid | ND | 0.39±0.01 | 0.05±0.01 | 0.09±0.01 | ND | 0.01±0.01 | 0.03±0.01 | 0.02±0.01 |
| Eicosanoid acid | 0.04±0.01 | 0.03±0.01 | 0.02±0.01 | 0.02±0.01 | 0.02±0.01 | 0.01±0.01 | 0.02±0.01 | 0.02±0.01 |
| Nucleic acid | | | | | | | | |
| Pyrimidine | ND | 0.03±0.01 | 0.03±0.01 | 0.02±0.01 | 0.07±0.01 | 0.03±0.01 | 0.05±0.01 | 0.03±0.01 |
| 4-Pyrimidinamine | ND | 0.09±0.01 | 0.81±0.01 | ND | ND | 0.06±0.01 | 0.06±0.01 | 0.09±0.01 |
| 9H-Purine | ND | ND | ND | ND | ND | ND | 0.08±0.01 | ND |
| 9H-Purin-6-amine | 0.05±0.01 | ND | 0.04±0.01 | 0.03±0.01 | 0.06±0.01 | 0.05±0.01 | 0.1±0.01 | 0.08±0.01 |
| Inosine | 0.02±0.01 | 0.02±0.01 | 0.01±0.01 | 0.01±0.01 | 0.1±0.01 | 0.01±0.01 | 0.04±0.01 | 0.07±0.01 |
| Adenosine | ±0 | ND | ND | ND | ND | ND | 0.02±0.01 | 0.02±0.01 |
| Amine | | | | | | | | |
| Tris(trimethylsilyl)  hydroxylamine | 0.50±0.17 | 0.30±0.06 | 0.32±0.02 | 0.37±0.06 | 0.29±0.06 | 0.25±0.03 | 0.20±0.04 | 0.38±0.06 |
| Urea | 0.05±0.01 | 0.04±0.01 | 0.04±0.01 | 0.29±0.01 | 0.05±0.01 | 0.04±0.01 | 0.04±0.01 | 0.04±0.01 |
| Cadaverine^1^ | 0.03±0.01 | 0.05±0.01 | 0.03±0.01 | 0.03±0.01 | 0.06±0.01 | 0.03±0.01 | 0.04±0.01 | 0.03±0.01 |
| Cadaverine^2^ | 0.13±0.04 | 0.06±0.01 | 0.09±0.01 | 0.08±0.01 | 0.11±0.02 | 0.07±0.01 | ND | ND |
| 1,4-Butanediamine | ND | ND | ND | ND | ND | ND | 0.08±0.01 | 0.07±0.01 |
| 1H-Indole-3-ethanamine | 10.84±3.10 | 5.81±1.01 | 7.51±0.49 | 6.87±0.79 | 9.41±1.10 | 6.52±0.29 | 5.86±0.28 | 5.74±0.22 |
| Cadaverine tri-TMS | 4.41±1.16 | 3.08±0.55 | 3.29±0.39 | 3.48±0.42 | 4.48±0.88 | 3.52±0.13 | 3.32±0.22 | 3.19±0.31 |
| 2-Thiopheneacetamide | ND | ND | ND | ND | 0.04±0.01 | 0.03±0.01 | 0.04±0.01 | 0.03±0.01 |
| Androst-2-en-17-amine | 3.48±0.15 | 1.78±0.18 | 1.91±0.15 | 1.78±0.19 | 2.41±0.15 | 1.78±0.14 | 1.86±0.11 | 1.79±0.19 |
| Others | | | | | | | | |
| 4-Hydroxypyridine | 0.10±0.01 | 0.06±0.01 | 0.06±0.01 | 0.06±0.01 | 0.08±0.01 | 0.08±0.01 | 0.17±0.01 | 0.07±0.01 |
| 1-Monolinoleoylglycerol | 0.05±0.02 | 0.03±0.01 | 0.03±0.01 | 0.03±0.01 | 0.05±0.01 | 0.03±0.01 | 0.03±0.01 | 0.03±0 |
| Phosphoric acid^1^ | ND | ND | ND | ND | 0.04±0.01 | 0.01±0.01 | 0.03±0.01 | 0.01±0.01 |
| Phenylethanolamine triTMS | 0.27±0.05 | 0.23±0.05 | 0.24±0.07 | 0.27±0.03 | 0.41±0.06 | 0.27±0.03 | 0.29±0.01 | 0.25±0.02 |
| Phosphonic acid | 0.13±0.01 | 0.08±0.02 | 0.05±0.01 | 0.07±0.01 | 0.05±0.08 | 0.15±0.02 | 0.11±0.02 | 0.11±0.02 |
| 2-(4-Methoxyp-henyl)-2-(4-tri-methoxysilyloxy) propane | 0.54±0.18 | 3.90±1.40 | 0.38±0.09 | 0.79±0.24 | 3.54±0.70 | 7.13±0.50 | 18.01±2.76 | 10.03±1.45 |
| Phosphoric acid^2^ | ND | ND | ND | ND | ND | 0.02±0.01 | 0.22±0.02 | 0.01±0.01 |
| Pregna-1,4,6-triene-3,20-dione | 0.81±0.13 | 0.41±0.13 | 0.31±0.04 | 0.37±0.04 | 0.51±0.01 | 0.41±0.07 | 0.40±0.02 | 0.32±0.02 |
| 9,10-Anthracenedione | 0.05±0.01 | 0.03±0.01 | 0.02±0.01 | 0.24±0.01 | 0.03±0.01 | 0.02±0.01 | 0.02±0.01 | 0.03±0.01 |
| 1,1,1,5,7,7,7-Heptamethyl  -3,3-bis (trimethylsiloxy  ) tetrasiloxane | 0.11±0.07 | 0.08±0.03 | 0.06±0.01 | 0.02±0.01 | 0.09±0.03 | 0.05±0.02 | 0.03±0.01 | 0.04±0.02 |
| 2-Monopalmitin | 0.16±0.04 | 0.08±0.02 | 0.06±0.01 | 0.06±0.01 | 0.11±0.03 | 0.06±0.01 | 0.06±0.01 | 0.09±0.01 |
| Estra-1,3,5(10)-trien-6-one | 0.35±0.05 | 0.18±0.06 | 0.17±0.03 | 0.18±0.04 | 0.21±0.03 | 0.17±0.03 | 0.12±0.01 | 0.15±0.02 |
| 2-Monostearin | 0.37±0.03 | 0.23±0.10 | 0.17±0.02 | 0.22±0.03 | 0.22±0.03 | 0.16±0.03 | 0.16±0.03 | 0.15±0.02 |
| Squalene | 0.24±0.06 | 0.15±0.05 | 0.26±0.03 | 0.16±0.03 | 0.14±0.02 | 0.12±0.03 | 0.08±0.01 | 0.16±0.05 |

^1, 2^ refers to the different configurations of the same substance; ND refers to no data was obtained

**TABLE S2.** Statistical data of *PCA* and *PLS*

| Model | R^2^X | R^2^Y | Q^2^ |
| --- | --- | --- | --- |
| PCA | 0.977 | - | 0.596 |
| PLS | 0.977 | 0.962 | 0.958 |

R^2^X and R^2^Y represent the accuracy of fit and Q^2^ represents the predictability of the model


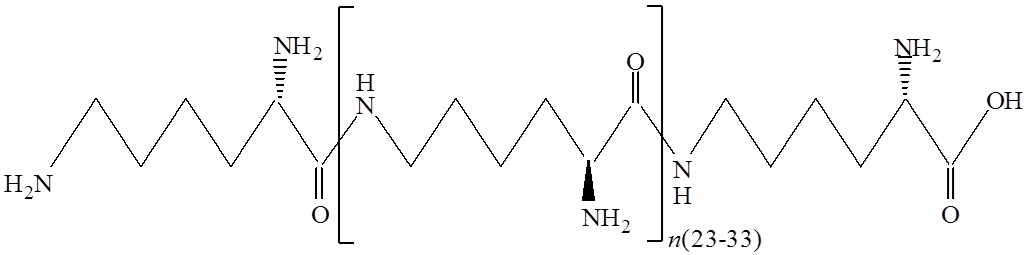


**FIGURE S1.** Schematic representation of the chemical structure of *ε-PL*


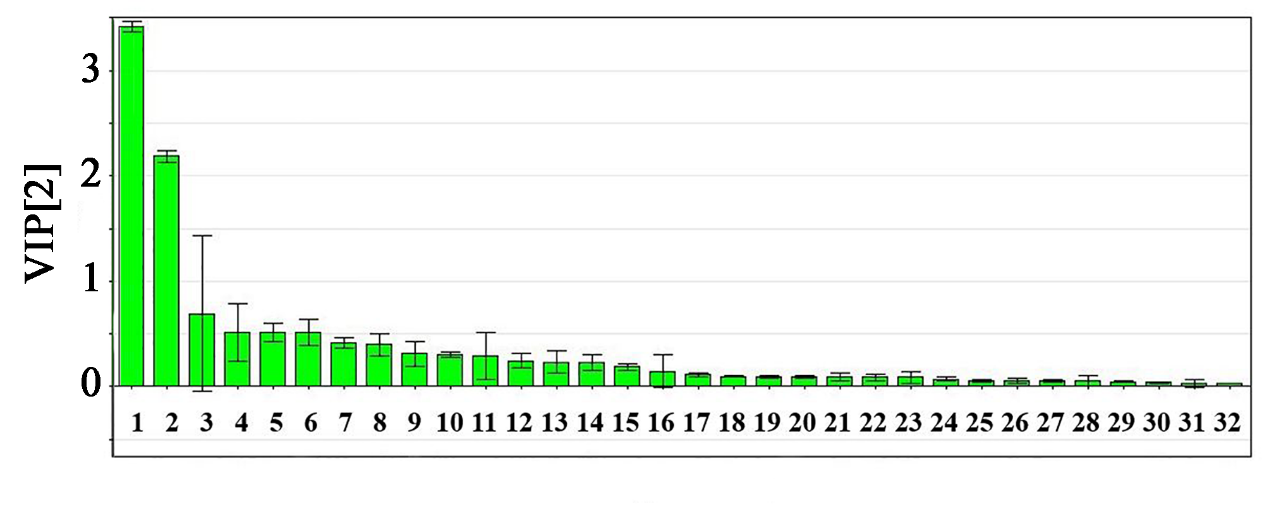


**FIGURE S2.** *VIP* results of differential metabolites, 1: D-Glucose; 2: D-Galactose; 3: D-(+)-Trehalose; 4: Stearic acid; 5: Palmitic acid; 6: Glycerol; 7: L-Alanine; 8: Oxalic acid; 9: Butanoic acid; 10: Octadecanoic acid; 11: Hexadecanoic acid; 12: Glutamic acid; 13: L-Proline; 14: Ethanediodic acid; 15: Glucopyranose; 16: N-α-acetyl-L-lysine; 17: Cadaverine tri-TMS; 18: Androst-2-en-17-amine; 19: Glycyl-l-glutamic acid; 20: D-Erythro-pentose; 21: L-Valine; 22: D-Xylopyranose; 23: β-D-Galactopyranoside; 24: Propanoic acid; 25: L-Threonine; 26: Pregna-1,4,6-triene-3,20-dione; 27: L-Isoleucine; 28: L-Phenylalanine; 29: Tetradecanoic acid; 30: L-Serine; 31: 2-Monostearin; 32: Adenosine


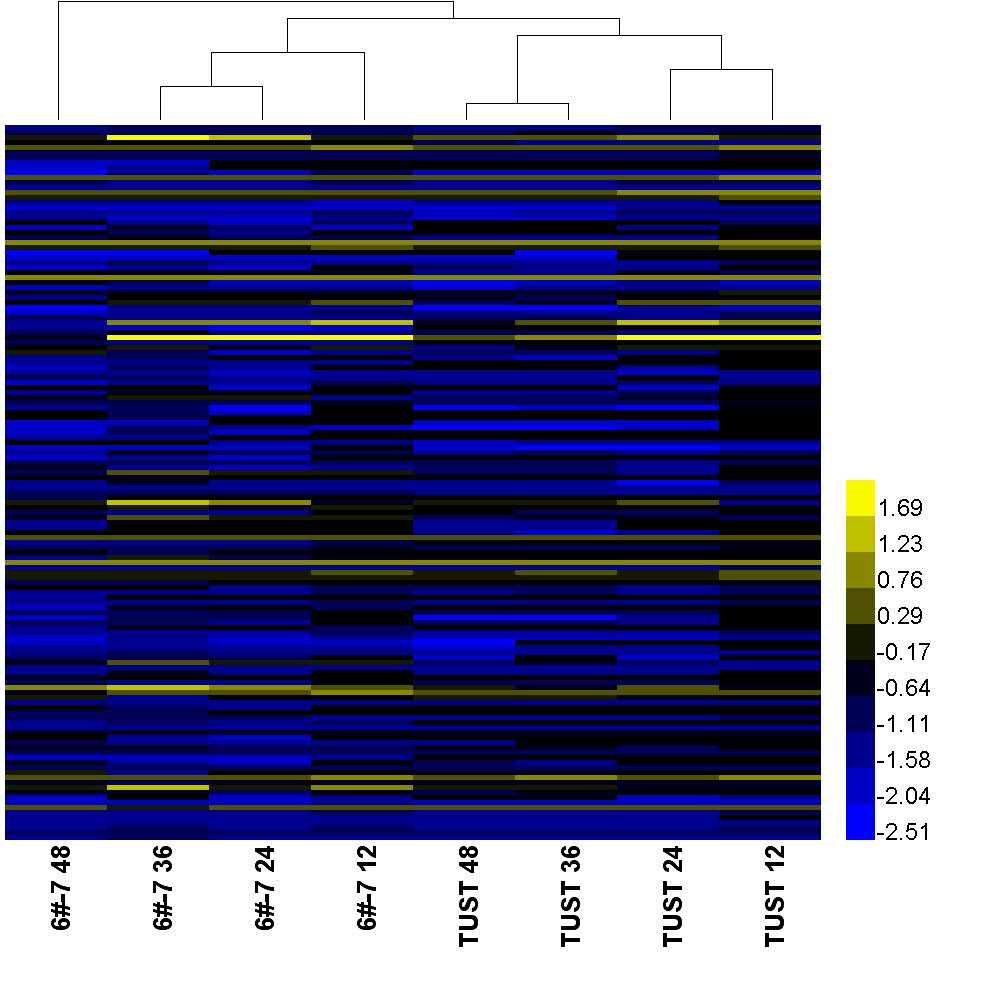


**FIGURE S3.** *HCA* results of the strains at different fermentation stages

**
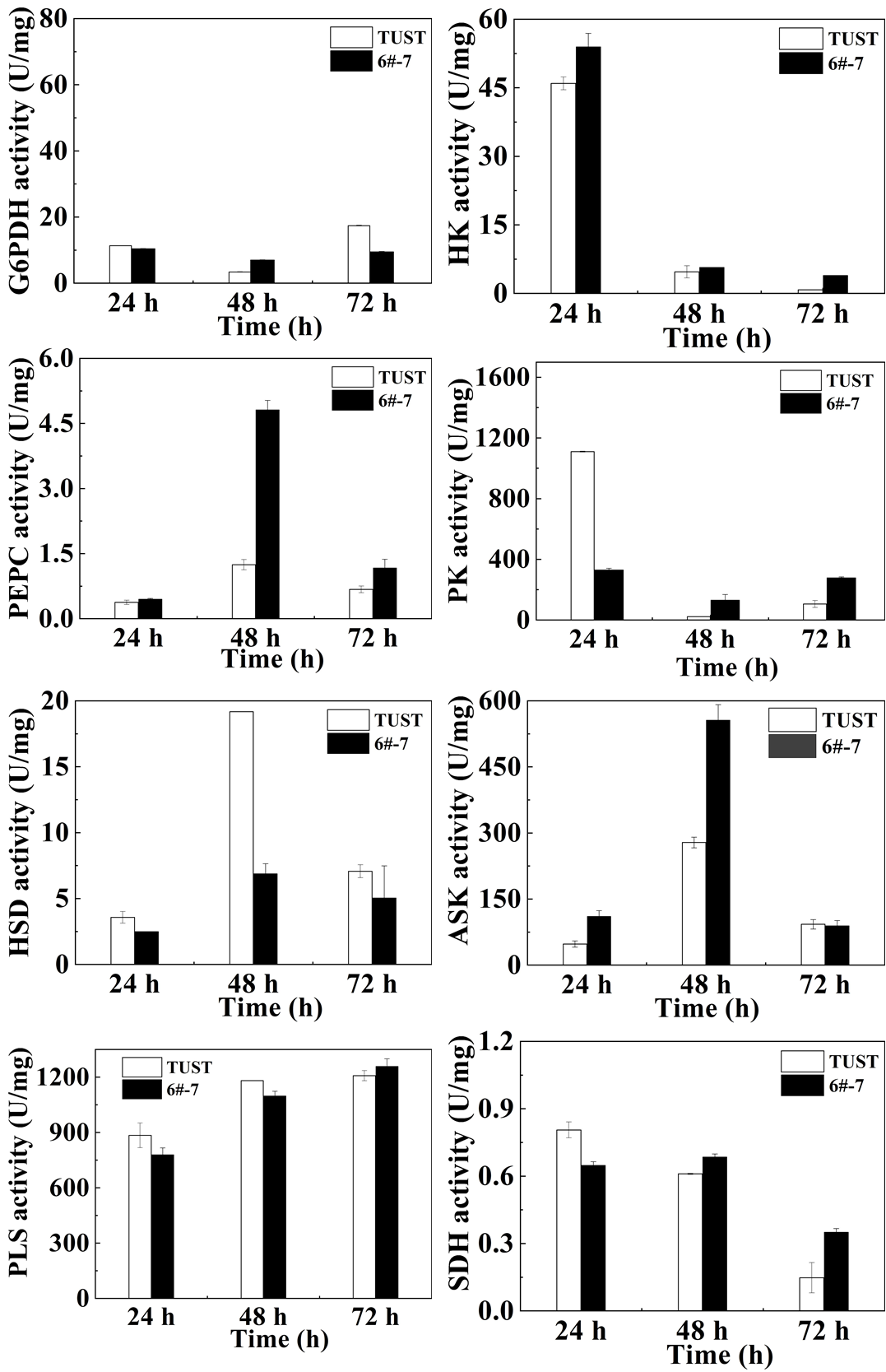
**

**FIGURE S4.** The activities of key enzymes. *HK*: Hexokinase; *PK*: Pyruvate kinase; *PEPC*: Phosphoenolpyruvate carboxylase; *ASK*: Aspartokinase; *HSD*: Homoserine dehydrogenase; *G6PDH*: Glucose-6-phosphate dehydrogenase; *SDH*: Succinate dehydrogenase; *PLS*: Polylysine synthetase
